# Exploring psychosocial predictors of STI testing in University students

**DOI:** 10.1186/s12889-018-5587-2

**Published:** 2018-05-29

**Authors:** H. A. Martin-Smith, E. A. Okpo, E. R. Bull

**Affiliations:** 1 NHS Grampian, Public Health Directorate, Aberdeen, UK; 2 Division of Medical Education, School of Medical Sciences, Faculty of Biology, Medicine and Health, The University of Manchester, Manchester, UK

**Keywords:** Sexual health, Students, Health promotion, Sexually transmitted infections, Testing, Psychosocial determinants

## Abstract

**Background:**

To explore university students’ Sexually Transmitted Infection (STI) testing knowledge, psychosocial and demographic predictors of past STI testing behaviour, intentions to have an STI test, and high risk sexual behaviour, to inform interventions promoting STI testing in this population.

**Methods:**

A cross-sectional, quantitative online survey was conducted in March 2016, recruiting university students from North East Scotland via an all-student email. The anonymous questionnaire assessed student demographics (e.g. sex, ethnicity, age), STI testing behaviours, sexual risk behaviours, knowledge and five psychological constructs thought to be predictive of STI testing from theory and past research: attitudes, perceived susceptibility to STIs, social norms, social fear and self-efficacy.

**Results:**

The sample contained 1294 sexually active students (response rate 10%) aged 18–63, mean age = 23.61 (SD 6.39), 888 (69%) were female. Amongst participants, knowledge of STIs and testing was relatively high, and students held generally favourable attitudes. 52% reported ever having an STI test, 13% intended to have one in the next month; 16% reported unprotected sex with more than one ‘casual’ partner in the last six months. Being female, older, a postgraduate, longer UK residence, STI knowledge, perceived susceptibility, subjective norms, attitudes and self-efficacy all positively predicted past STI testing behaviour (*p* < 0.01). Perceived susceptibility to STIs and social norms positively predicted intentions to have an STI test in the next month (*p*  < 0.05); perceived susceptibility also predicted past high-risk sexual behaviour (*p* < 0.01).

**Conclusions:**

Several psychosocial predictors of past STI testing, of high-risk sexual behaviour and future STI intentions were identified. Health promotion STI testing interventions could focus on male students and target knowledge, attitude change, and increasing perceived susceptibility to STIs, social norms and self-efficacy towards STI-testing.

**Electronic supplementary material:**

The online version of this article (10.1186/s12889-018-5587-2) contains supplementary material, which is available to authorized users.

## Background

Sexually Transmitted Infections (STIs) are a significant public health problem. In England there were nearly half a million new diagnoses of STIs in 2015 alone [1]. If not treated, STIs can lead to serious long-term health sequelae such as pelvic inflammatory disease and infertility [1]. The impact of STIs is greatest in young people under 25 years old [1], including university students, who, are most likely to be exposed to key risk factors such as multiple sexual partners and unprotected sexual intercourse [2]. When used correctly and consistently, condoms are the most effective means of preventing an STI. Prompt testing, early diagnosis and treatment of STIs can reduce the incidence and complications associated with the disease by reducing onward transmission of infection to sexual contacts [3, 4].

Proactive approaches to STI testing tend to use population registers to invite individuals from a target population for screening; in opportunistic approaches individuals are offered testing whilst attending health services for other reasons [5]. Some European countries have national STI testing programmes, however uptake rates are generally low [6]. Results from a recent large survey in the UK [7] suggested that although rates of chlamydia testing in young adults aged 16–25 are increasing [8], the level still falls below the 35% of population needed to reduce prevalence of STIs [9]. Many young people are not attending STI testing services and so effective efforts to encourage testing uptake are required.

To date a limited number of studies have explored the psychosocial determinants of STI testing in a university setting. Traditional STI research has shown that STI testing rates can vary substantially according to age, gender, ethnicity, education level as well as STI-related knowledge and systemic factors [10]. More recent research has explored more complex social and psychological factors such as perceived norms and perceived susceptibility informed by health psychology theories [11–13] and found these to be significant predictors of behaviour.

Indeed, according to the Theory of Planned Behaviour [14] a person’s behaviour depends on their ‘behavioural beliefs’, or attitudes, relating to possible outcomes of the behaviour, their ‘normative beliefs’ about significant others’ expectations about their behaviour, which can create perceived social pressure (subjective norms) and ‘control beliefs’ about the power of barriers to performing the behaviour, which creates perceived behavioural control. Positive attitudes, subjective norms and perceived behavioural control lead to stronger intention and thus likelihood of behaviour change. This theory has been applied to explain sexual behaviours such as condom use [15] and more recently in understanding barriers to STI testing [12]. Later versions of the model and meta-analyses also acknowledge the additive effect of descriptive norms, or individuals’ beliefs about what others’ do, which may be especially important for younger populations and for health risk behaviours [16, 17].

The Health Belief Model [18] proposed alternative constructs including perceived susceptibility or vulnerability to a condition (such as an STI), perceived severity if it occurred, perceived benefits of taking preventative action (e.g. STI testing) and beliefs about barriers to this action. This model has also been applied to explain condom use [19] and only recently to understanding the factors impacting a person’s decision to take an STI test [12].

Social Cognitive Theory [20] emphasises self-efficacy (one’s confidence to perform behaviour) in determining which behaviour a person chooses to perform, their persistence in the face of obstacles and how well they will perform that behaviour. Increased self-efficacy is also thought to be one mediator of the link between descriptive norms and behaviour, the idea that ‘if similar people to me do X, then I must be able to do it too’ [21]. The role of self-efficacy has been applied to explain the initiation and maintenance of many health behaviours including alcohol consumption [22]; physical activity [23], and many others; again infrequently in STI testing [12].

In addition social factors (such as social fear based on sociological research of stigma and fear of negative consequences) has been identified to impact on a young persons decision to have an STI test from qualitative and quantitative research [12, 24].

Application of these theories and sociological factors in exploring STI testing behaviours have resulted in somewhat inconsistent and fragmented findings [11–13]. Furthermore, sexual health promotion interventions and communications are not always based on careful analysis of the evidence around predictors of behaviour: one study of safer sex leaflets found most messages targeted knowledge about condoms rather than information about perceived attitudes even though the latter was more strongly correlated with actual condom use [25].

Building on this evidence is important to gain a further understanding of social and psychological factors influencing STI testing behaviour, particularly to inform practice for those working in settings with high proportions of under 25 year olds, such as universities. The objectives of this study were to:

1. Explore university students’ STI testing knowledge, psychosocial and demographic factors and behaviours.
2. Understand which psychosocial and demographic factors predict STI testing, intentions and sexual risk behaviour.

Ultimately, this aimed to inform effective interventions for promoting STI testing in university students.

## Methods

### Design

A quantitative cross-sectional survey study using an anonymous, self-administered online survey developed following a review of the literature. The questionnaire was developed systematically using theory-based psychological constructs found to be predictive of behaviour in previous research, and where possible, using validated measures and scores (see Additional file 1 for further detail on definitions and measures and Additional file 2 for the full questionnaire). Questions assessed students’ sexual health behaviours, intentions to attend an STI test in the next month, demographic characteristics (age, sex, ethnic background, year of study and number of years living in the UK), STI knowledge and the five theory-based psychosocial constructs (perceived susceptibility, social norms, attitudes, social fear and self-efficacy). The questionnaire was piloted with a convenience sample of seven health psychology researchers and students and minor amendments were made. The main behavioural outcome variables were STI testing history (ever), intentions to attend an STI test in the next month and sexual risk behaviour (defined in this study as unprotected (no condom) sex with two or more sexual partners in the last six months).

### Setting

This study was conducted among students in a North East Scotland University in March 2016.

### Participants

Eligible participants were all sexually active registered undergraduate and postgraduate students in all faculties in a North East Scotland University in March 2016 (16,435 students).

Participants were invited to complete the survey via an email that was sent to all the students from the university with a link to the online survey questionnaire. Participation was voluntary, anonymous and required online consent. Students were introduced to the topic and advised of the voluntary and anonymous nature of the study, their right to withdraw, and to researcher contact details. An opt-in checkbox indicated participants’ informed consent. Only participants who indicated that they had ever been sexually active were included in the final analysis. The survey was live for two weeks, during which time two reminder emails were sent to facilitate recruitment.

### Statistical analyses

The submitted anonymous surveys were coded numerically, scores for individual items were summed into composite scores based on previous research (full details of data treatment can be found in Additional file 1), and analysis was performed using SPSS (version 20.0).

To further explore sample characteristics in relation to population characteristics, we examined differences between our sample with the university student population, using demographic data on ethnicity, age and sex from the University records office, using Chi-square tests.

Data were assessed for normal distribution using Shapiro-Wilk test of normality. Where data were not normally distributed, the median and interquartile range (IQR) was reported. Chi-square tests and Mann Whitney U tests examined differences, means ranks were investigated when differently shaped distributions of the independent variable occurred. Odds Ratios (OR) with associated 95% confidence intervals (95% CI) were used to measure the association between two attributes. Logistic regression models were then used to assess which psychosocial (including knowledge and demographic) factors predicted past STI testing, sexual risk behaviour and intentions to attend STI testing in the next month. Cronbach’s alpha was used to assess the internal consistency of items within each construct of psychosocial measures, with >.07 taken as adequate consistency. Cronbach alpha coefficients were all above 0.7 except for the descriptive norms scale [α = .59]. There was poor inter-item correlation between the three question items (*r* = 0.11–0.52) making up this scale and little improvement in Cronbach’s alpha with the deletion of any item, so for parsimony descriptive norms was removed from the analysis.

## Results

### Participants

A total of 1600 students participated (response rate 10%), 297 were excluded due to sexually inactivity and 9 were excluded owing to not completing over 50% of the survey. The final analyses were performed on 1294 individuals. Of these, 69% (888/1294) were female and the mean age was 23.68 years (standard deviation, SD 6.44, range 18–63). The response rate was higher for female undergraduates from a white ethnic background compared to official university records on the overall female student population. Table 1 shows the characteristics of study participants.

**Table 1:** Participant characteristics and median and interquartile range scores for each psychosocial variable

Demographics					*Psychosocial variablesMedian (IQR)*						
		*Overall N(%)*	Overall Median (IQR)	Knowledge	Susceptibility	*Subjective norms*	*Descriptive norms*	*Direct Attitudes*	*Indirect attitudes*	*Social fear*	*Self-Efficacy*
Age	18–25	999 (77)	22 (20–25)	11.0 (9.0–12.0)	4.0 (4.0–5.0)	*5.0 (12.0–22.0)*	*8.0 (6.0–9.0)*	*31.0 (28.0–33.0)*	*42.0 (39.0–46.0)*	*17.0 (11.0–25.0)*	*38.0 (31.0–46.0)*
	25 or more	282 (21)		11.0 (8.0–12.0)	4.0 (4.0–5.0)	*0.0 (−20.0–18.5)*	*7.0 (6.0–9.0)*	*31.0 (28.0–32.0)*	*43.0 (38.0–46.0)*	*15.0 (10.0–22.0)*	*38.0 (32.0–48.0)*
Sex	Male	401 (31)	/	11.0 (8.0–13.0)	4.0 (4.0–5.0)	4.0 (−12.0–19.0)	7.0 (6.0–9.0)	30.0 (27.0–32.0)	42.0 (38.0–45.0	16.0 (10.0–23.0)	*38.0 (33.0–48.0)*
	Female	888 (69)		10.0 (9.0–12.0)	4.0 (4.0–5.0)	*4.0 (−16.0–22.0)*	*8.0 (7.0–9.0)*	*31.0 (29.0–33.0)*	43.0 (39.0–46.0)	*17.0 (10.0–25.0)*	*37.0 (31.0–46.0)*
Ethnicity	White	1129 (87)	/	11.0 (9.0–12.0)	4.0 (4.0–5.0)	*5.0 (−14.0–22.0)*	*8.0 (7.0–9.0)*	*31.0 (28.0–32.0)*	*42.0 (39.0–46.0)*	16.0 (10.0–24.0)	*38.0 (32.0–47.0)*
	Mixed Multiple	23 (2)		9.0 (6.0–12.0)	4.0 (4.0–5.0)	*0.0 (−16.0–14.5)*	7.0 (6.0–8.5)	*31.0 (27.0–33.0)*	43.0 (38.0–47.0)	*17.0 (10.0–24.5)*	*36.0 (28.0–43.0)*
	Asian	73 (6)									
	Black	*n* < 5									
	African	31 (2)									
	Other	30 (2)									
Years in UK	0–5	456 (35)	19 (3.0–21.0)	9.0 (7.0–11.0)	4.0 (4.0–5.0)	*0.0 (− 15.0–20.0)*	7.0 (6.0–9.0)	*31.0 (29.0–33.0)*	43.0 (39.0–47.0)	*15.0 (9.5–23.0)*	*38.0 (31.0–47.0)*
	More than 5	838 (65)		11.0 (9.0–11.0)	4.0 (4.0–6.0)	6.0 (−14.0–22.0)	*8.0 (7.0–9.0)*	*31.0 (28.0–32.0)*	*42.0 (38.0–45.0)*	*17.0 (11.0–25.0)*	*38.0 (32.0–46.0)*
Year of study	Undergraduate	968 (75)	/	11.0 (9.0–12.0)	4.0 (4.0–6.0)	*5.0 (−13.0–22.0)*	*8.0 (7.0–9.0)*	*31.0 (38.0–32.0)*	*42.0 (39.0–46.0)*	*17.0 (11.0–24.0)*	*38.0 (23.0–46.0)*
	Post graduate	325 (25)		10.0 (7.0–12.0)	4.0 (4.0–5.0)	*0.0 (−17.0–17.0)*	7.0 (6.0–9.0)	*31.0 (28.0–33.0)*	43.0 (38.0–46.0)	16.0 (10.0–24.0)	*36.0 (31.0–47.0)*
	Overall Median (IQR)			10.0 (9.0–12.0)	4.0 (4.0–5.0)	4.0 (−15.0–20.0)	*8.0 (6.0–9.0)*	*31.0 (28.0–32.0)*	*42.0 (39.0–46.0)*	16.0 (10.0–24.0)	*38.0 (31.0–47.0)*
	Range of possible scores			0–14	4–20	−40 − + 40	*3–15*	*8–35*	*11–55*	*8–40*	*12–60*

#### Objective 1: Student knowledge, psychosocial and demographic factors surrounding STI testing

##### Knowledge of STIs/STI-testing

In the 14-item (true/false) STI/STI-Testing knowledge section, the median number of correctly answered questions was 10.0 (IQR 9.0–12.0). Male students (median = 11.0, IQR = 8.0–13.0 had greater STI/STI-Testing knowledge than female students (median = 10.0, IQR = 9.0–12.0) however this was not found to be a statistically significant difference (U = 174,242.5, z = −.619, *p* = .53). Similarly white students (median = 11.0, IQR = 9.0–12.0), students who had lived in the UK more than 5 years (median = 11.0, IQR = 9.0–11.0) and undergraduate students (median = 11.0, IQR = 9.0–12.0) had greater STI/STI-Testing knowledge than non-white students (median = 9.0, IQR = 6.0–12.0), students who had lived in the UK 0–5 years (median = 9.0, IQR = 7.0–11.0) and postgraduate students (median = 10.0, IQR = 7.0–12.0), and these differences were found to be statistically significant (*p* < .001).

##### Psychosocial factors surrounding STIs and STI testing

Overall, participant direct and indirect attitudes were favourable towards STI testing (Median = 31.0, IQR = 28.0–32.0 and Median = 42.0, IQR = 39.0–46.0 respectively). In terms of the other psychosocial variables, social fear towards STI testing and perceptions of susceptibility to an STI were low (Median = 16.0, IQR = 10.0–24.0) and (Median = 4.0, IQR = 4.0–5.0) respectively. Perception of social pressure (subjective norms) and confidence (self-efficacy) to attend an STI test were moderate (Median = 4.0, IQR = − 15.0–20.0) and (Median = 38.0, IQR = 31.0–47.0 15.0) respectively. The median score for descriptive norms towards STI testing was moderate (Median = 8.0, IQR = 6.0–9.0). Table 1 includes median and interquartile ranges for participants’ scores on the psychosocial variables.

##### Behaviour

Fifty two percent (678) of students who completed the survey reported having ever had an STI test, 20.5% (266) reported having had an STI test in the last six months, 13% (166) indicated an intention to attend an STI test in the next month and 16% (181) reported unprotected sex with more than one casual partner in last six months.

#### Objective 2: Psychosocial factors predicting university students’ past STI testing behaviours, intentions to attend an STI test and sexual risk behaviours

##### Past STI testing

Results of regression analysis showed that females and students aged 25 or over, postgraduates and students who had been in the UK for more than 5 years were more likely to have had an STI test in the past. Similarly knowledge, perceived susceptibility, subjective norms, direct and indirect attitudes and self-efficacy were all predictors of past STI testing behaviour. High scores on these variables meant students were more likely to have had an STI test in the past (Table 2).

**Table 2:** Results of logistic regression of past STI testing behaviour (*n* = 1212)

	Variable		Odds ratio	95% CI	*p value*
Demographics	Age	18–25	Reference		
		25 or more	1.522	1.019–2.273	*p = .040*
	Sex	Male	Reference		
		Female	2.134	1.599–2.847	*p < .001*
	Ethnicity	Non White	Reference		
		White	1.107	0.718–1.796	*p = .645*
	Number of years in UK	0–5	Reference		
		More than 5	0.706	0.521–0.957	*p = .025*
	Year of study	Undergraduate	Reference		
		Post graduate	1.551	1.053–2.285	*p = .026*
Psychosocial	Knowledge		1.254	1.185–1.326	*p < .001*
	Susceptibility		1.076	1.022–1.134	*p = .006*
	Subjective norms		1.014	1.008–1.020	*p < .001*
	Direct attitudes		1.037	1.000–1.075	*p = .048*
	Indirect attitudes		1.063	1.030–1.097	*p < .001*
	Social Fear		1.000	0.982–1.018	*p = .972*
	Self-Efficacy		1.042	1.027–1.057	*p < .001*
	Intention		1.027	0.667–1.581	*P = .901*

##### Intentions to go for an STI test

Furthermore, ethnicity, perceived susceptibility and social norms predicted intentions to get tested in the next month with white students being less likely to express an intention to get an STI than those of other ethnicities, and high perceived susceptibility and subjective norms meaning students were more likely to express intention to get tested (Table 3).

**Table 3:** Results of logistic regression of intentions to have an STI test in the next month (*n* = 1212)

	Variable		Odds ratio	95% CI	*p value*
Demographics	Age	18–25	Reference		
		25 or more	1.446	0.825–2.536	*p = .198*
	Gender	Male	Reference		
		Female	0.889	0.590–1.340	*p = .575*
	Ethnicity	Non White	Reference		
		White	0.425	0.246–0.735	*p = .002*
	Number of years in UK	0–5	Reference		
		More than 5	0.913	0.588–1.417	*p = .685*
	Year of study	Undergraduate	Reference		
		Post graduate	0.637	0.355–1.141	*p = .129*
Psychosocial	Knowledge		1.005	0.927–1.090	*p = .901*
	Susceptibility		1.236	1.174–1.300	*p < .001*
	Subjective norms		1.024	1.015–1.034	*p < .001*
	Direct attitudes		1.052	0.990–1.118	*p = .100*
	Indirect attitudes		1.030	0.985–1.078	*p = .194*
	Social Fear		1.002	0.976–1.030	*p = .869*
	Self-Efficacy		1.007	0.987–1.028	*p = .484*
	Past STI testing		1.078	0.696–1.670	*p = .735*

##### Sexual behaviour

Table 4 below shows that socio-demographic variables had no statistically significant effect on high risk sexual behaviour in the past six months, defined as having had unprotected (no condom) sex with multiple sexual partners (two or more) in the last six months. Perceived susceptibility and subjective norms were the only predictors of past risky behaviour. For every one-unit increase in susceptibility and subjective norms score, students were 33 and 1% more likely to have engaged in risky behaviour in the past, respectively.

**Table 4:** Results of logistic regression of sexual risk behaviour i.e. Unprotected (no condom) and multiple sexual partners (2 or more) in the last 6 months (*n* = 1104)

	Variable		Odds ratio	95% CI	*p value*
Demographics	Age	18–25	Reference		
		25 or more	0.656	0.355–1.212	*p = .178*
	Sex	Male	Reference		
		Female	0.854	0.574–1.269	*p = .434*
	Ethnicity	Non White	Reference		
		White	0.579	0.324–1.034	*p = .065*
	Number of years in UK	0–5	Reference		
		More than 5	1.243	0.801–1.930	*p = .333*
	Year of study	Undergraduate	Reference		
		Post graduate	0.591	0.325–1.073	*p = .084*
Psychosocial	Knowledge		1.044	0.965–1.130	*p = .284*
	Susceptibility		1.331	1.258–1.408	*p < .001*
	Subjective norms		1.011	1.002–1.019	*p = .018*
	Direct attitudes		1.001	0.950–1.054	*p = .974*
	Indirect attitudes		1.009	0.966–1.054	*p = .684*
	Social Fear		1.012	0.987–1.039	*p = .348*
	Self-Efficacy		1.007	0.987–1.028	*p = .488*

## Discussion

This study assessed knowledge, psychosocial and demographic factors and behaviours surrounding STI testing and explored theoretically-relevant psychosocial predictors of past STI testing, intentions to attend an STI test and high risk sexual behaviour in a student population in Scotland, United Kingdom.

Participants had a relatively high level of knowledge of STIs and STI testing and younger students (18–25 years), undergraduate and students who have lived in the UK for over 5 years statistically significantly answered more knowledge questions correctly.

Approximately half of participants reported ever attending an STI test, similar to the proportion of women reporting having had a chlamydia test in the national survey [7]. Similar to other studies, women and students over 25 years old were more likely to report having been tested for STIs [12], confirming that under 25 s may indeed be a ‘high-risk’ group. Women over 25 in particular may generally have more opportunity for STI testing given cervical screening and other contact with health care providers for reproductive health issues. Postgraduates and students who had lived in the UK for more than 5 years were also more likely to have had an STI test. Our study showed that knowledge was also a predictor of past STI testing behaviour. Similar findings have been reported by some studies [26, 27]. In contrast to one study in Australia [12] that reported no link between knowledge of STI and risk behaviour. A growing consensus suggests that increasing knowledge is necessary but not sufficient for behaviour change, given other complex factors that contribute to health-seeking behaviour (e.g. self-efficacy, social norms and socio-structural factors) [28]. Other less strongly positive associated predictors of STI testing included high perceptions of susceptibility, high social pressure (subjective norms) to attend STI testing, with favourable direct and indirect attitudes and high confidence to attend a test. These findings are inconsistent with previous studies, perhaps due to differences in methodology and culture. It emphasises the complex nature of sexual health behaviours such as STI testing cementing the need for diverse initiatives to tackle this public health problem.

Few students intended to attend an STI test in the next month (13%). Similar to other studies, intention to participate in STI testing among students in this population was driven by perceived susceptibility and subjective norms [11–13] and multiple partners [29] however other research [30, 31] identified variables such as perceived behavioural control, self-identity, motivation and cues to action as important predictors of intentions. This highlights the possible need to further explore intentions within this population including variables not assessed in the survey.

A small number of students reported engagement in sexual risk behaviour in the past (16%).

The finding that high scores of susceptibility for an STI and social pressure to attend an STI test meant students were more likely to have engaged in past risky behaviour is important. It suggests students accurately judged themselves to be susceptible, whilst feeling social pressure to attend an STI test if they’d taken a risk. In comparison previous reviews, explored determinants of sexual risk behaviour, but with inconsistent results. Some identified attitudes, behavioural intentions and behavioural skills as correlates of sexual behaviours [32]. The majority of this research was based on correlation data from cross-sectional studies which is limited as temporality is unable to be established. In light of the above, current theoretical models of sexual risk behaviour warrant further investigation and possible adaptation.

Since a range of psychosocial and demographic factors were associated with STI testing, sexual health promotion interventions could benefit from targeting specific student groups and by addressing relevant psychosocial factors. Some recommendations that interventions may address in their design are as follows 1) promote and strengthen positive norms around testing to build on health promotion, 2) address young people’s perceptions of personal risk of contracting an STI, 3) promote and strengthen positive attitudes towards STI testing, 4) increase confidence to attend STI testing. There are several practical intervention ideas based on previous research that could be applied to focus on these key psychosocial factors to increase uptake of STI testing. Firstly marketing campaigns that deliver health messages have been found to positively impact on changing health behaviours [33]. Peer-led screening uses peer education to promote positive social norms and positive attitudes through modelling and normalising [34]. A role-modelling intervention could involve displaying video messages around campuses with students sharing their experiences of going for an STI test to increase self-efficacy and finally utilising social networks within universities by recruiting advocates who could spread behaviour change recommendations to their peers. Furthermore since younger, undergraduate, male students were found to be less likely to have attended testing, it is feasible that these interventions could focus on this sub group of students to help encourage more young people to have an STI test.

Overall, this cross-sectional study contributes to the body of evidence on factors that impact STI testing behaviours and demonstrates the complexity of social and psychological factors that impact on STI testing behaviours; further research to validate the findings using a prospective longitudinal study is required.

### Strengths and limitations

The current study is the first of its kind in this population in Scotland, adding to the limited body of evidence in this topic area. It is strengthened by the systematic development of the survey allowing for a wide range of factors to be assessed including demographic, behavioural and psychosocial factors regarding STI testing. Moreover this study attracted a relatively large sample size comparing favourably with other small-scale cross-sectional studies. However there are several limitations, not least that the cross-sectional design allows only for statistically significant associations between behaviour and their potential determinants. Cross-sectional surveys use self-reports of behaviour, risking recall and reporting bias. Participation bias may also have occurred if some people may be more willing than others to participate in this sensitive topic, and given the online nature of the survey although most if not all students would be likely to be able to access a computer. Like many other surveys, males aged 18–25 were under-represented, which could affect validity and generalizability of the findings. Ceiling and floor effects occurred when measuring some of the psychosocial variables, limiting the range of data reported leading to non-normally distributed data. Fortunately, the analytical test used in this study did not require normally distributed data, and as the sample size in this study was fairly large the negative effect of this response bias is reduced. The 10% response rate was larger than the 7% reported in another university-based sexual health study [35]: other comparable online sexual health questionnaire studies have not tended to report response rates and yield smaller overall sample sizes [13, 36]. Our response rate was naturally smaller than the more representative UK-wide national survey The National Survey of Sexual Attitudes and Lifestyles [7], who are resourced to visit potential participants in their home and offer monetary incentives for participation. We were not able to assess the predictive effect of descriptive norms, despite this being a potentially important influence on young people’s behaviour [17] as internal consistency was poor with low inter-item correlation. Further exploratory work would be useful to better understand which are the key referent groups for university students, if descriptive norms vary substantially. Nevertheless, this study adds to our understanding of several theory-based psychological factors associated with high-risk behaviours and STI testing in this important student population: further replication on a national scale would now be useful.

## Conclusions

This study found several demographic and psychosocial factors influencing STI testing behaviour, high-risk sexual behaviour and future intentions towards STI testing. Perceptions of risks and social pressure are important factors in sexual risk behaviours in the past and intentions to attend an STI test in the future. STI testing interventions could target young, male students from outside the UK and aim to boost (increase) susceptibility (i.e. perceptions of vulnerability), social norms (i.e. perceptions of social pressure) and confidence to attend for testing. The results from this study provide a useful direction for further behavioural change research and health promotion intervention design.

## Additional files

Additional file 1: Definitions and measurements. This file provides further detail on measures and scoring for the questionnaire constructs. (DOCX 16 kb)

Additional file 2: STI Screening Questionnaire. This file provides the full questionnaire used to survey participants. (DOCX 76 kb)

## Abbreviations

CI: Confidence Interval
IQR: Interquartile Range
OR: Odds Ratio
SD: Standard Deviation
STI: Sexually Transmitted Infection
