# Supplementary material for: Exploring psychosocial predictors of STI testing in University students
Source: BMC Public Health. 2018 May 29;18:664. doi: 10.1186/s12889-018-5587-2 (PMC5975527; doi:10.1186/s12889-018-5587-2)
Supplement: Supplementary file 1 — Definitions and measurements. This file provides further detail on measures and scoring for the questionnaire constructs. (DOCX 16 kb) [file 12889_2018_5587_MOESM1_ESM.docx]

Additional file 1

***Definitions and measurements***

*Behaviours*

Sexual activity was assessed by two variables, where participants were asked to indicate if they had ever been sexually active (to assess their eligibility) and if they had been sexually active in the last 6 months. Number of sexual partners was assessed by one variable, where participants were asked to report number of sexual partners in the last 6 months. Past STI testing history was assessed by one variable, where participants were asked if they had ever bed tested for an STI with a choice of three options (yes but not in last 6 months, yes in the last 6 months and no). Intentions were assessed by one variable where participants were asked to indicate their intention to attend an STI test in the next month. Sexual risk behaviour was assessed by merging two variables; unprotected sexual activity using 4 items where participants were asked to indicate if they had ever engaged in unprotected (i.e. no condom vaginal /anal sex with a casual or regular partner) in the last 6 months and multiple sexual partners (2 or more) in the last 6 months into a single dichotomous variable.

*Knowledge*

STI knowledge was assessed using 14 true/false statements relating to the transmission, symptoms and prevention of STIs. Each correct answer scored one, all other responses, or a ‘don’t know’ response, scored zero. Item scores were summed to create a composite score which ranged from 0 to 14, where 0 to 4 represents low knowledge of STIs, 5 to 9 represents moderate knowledge and 10 to 14 represents high knowledge.

*Attitude*

Attitudes were assessed in two domains. Direct attitudes i.e. affective attitudes to STI testing were measured using 8 bipolar adjectives, which were evaluative (e.g.” waste of time” to “valuable”). Item scores were summed to create a composite score which ranged from 8 to 35, where 8 to 16 represents a negative attitude to STI testing, 17 to 26 represents a moderate attitude to STI testing and 27 to 35 represents a positive attitude to STI testing. Indirect attitudes i.e. behavioural beliefs of the perceived consequences of going STI testing, were measured using scores from an 11 item scale (e.g. “I will stay healthy”) where responses ranged from ‘unlikely to ‘likely’. Item scores were summed to create a composite score which ranged from 11 to 55, where 11 to 25 represents negative perceptions of the consequences of STI testing, 26 to 40 represents moderate perceptions of the consequences of STI testing and 41 to 55 represents positive perceptions of the consequences of STI testing.

*Susceptibility*

Perceived susceptibility was assessed using 4 items with any STI, chlamydia, gonorrhoea and genital herpes (e.g. “Considering your answers and what you know about STIs what do you think your chances are of contracting an STI?”) (Based on previous research^13^ ). Item scores were summed to create a composite score which ranged from 4 to 20, where 4 to 9 represents low susceptibility to contracting an STI, 10 to 14 represents moderate susceptibility contracting an STI and 15 to 20 represents high susceptibility contracting an STI.

*Social norms*

Social norms were assessed in two domains. Firstly a 3 item scale of descriptive norms i.e. people's perceptions of others’ STI testing behaviour with reference to 3 groups: partners, friends, family and (e.g. “what proportions of your friends have ever been tested for STIs?” adapted from previous scales.^13^ Item scores were summed to create a composite score which ranged from 3 to 15, where 3 to 6 represents low perceptions of others getting STI tests, 7 to 10 represents moderate perceptions of others getting STI tests and 11 to 15 represents high perceptions of others getting STI tests. Secondly an 8 item scale of subjective norms i.e. perceived social pressure to perform or not perform a behaviour^14^ in this case in relation to attending an STI test with reference to 4 groups: partners, friends, family and health professionals. Items for subjective norms were adapted from previous research, and were made up of 4 items of normative beliefs (i.e. perceptions of significant others’ preferences about whether one should go STI testing, e.g. “My friends would want me to be tested for STIs”) and 4 items of motivation to comply with these (e.g. If I knew my friends wanted me to get tested for STIs I would...”).^13^ For each normative belief, the belief score on the strongly disagree to strongly agree scale is multiplied by the score relating to the motivation to comply (definitely be tested/definitely be tested) scale and summed to create an overall composite score. Scores ranged from -40 to +40 where -40 to -13 represents low perceptions of social pressure to go for an STI test, -13 to 13 represents moderate perceptions of social pressure to go for an STI test and 13-40 represents high perceptions of social pressure to go for an STI test.

*Social fear*

Eight items adapted from previous research assessed social fear (e.g. “If I had an STI people would think badly of me).^12^ Item scores were summed to create a composite score which ranged from 8 to 40, where 8 to 19 represents a low perception of social fear towards STI testing, 20 to 29 represents a moderate perception of social fear towards STI testing and 30 to 40 represents a high perception of social fear towards STI testing.

*Self-efficacy*

Self-efficacy was measured using 12 items (e.g. “How confident are you to get an STI test if the test site was far away?”. Item scores were summed to create a composite score which ranged from 12 to 60, where 12 to 27 represent low self efficacy to get STI tested, 28 to 44 represent moderate self-efficacy to get STI tested and 45 to 60 represent high self-efficacy to get STI tested.
