# Supplementary material for: Exploring psychosocial predictors of STI testing in University students
Source: BMC Public Health. 2018 May 29;18:664. doi: 10.1186/s12889-018-5587-2 (PMC5975527; doi:10.1186/s12889-018-5587-2)
Supplement: Supplementary file 2 — STI Screening Questionnaire. This file provides the full questionnaire used to survey participants. (DOCX 76 kb) [file 12889_2018_5587_MOESM2_ESM.docx]

*STI Testing Questionnaire*

**PART 1: DEMOGRAPHICS AND SELF-REPORTED SEXUAL HEALTH AND STI TESTING BEHAVIOUR**

Please answer the following questions:

What is your age?

o ______

Which best describes your sex?

o Female oMale

What is your ethnic group? (Choose one option that best describes your ethnic group or background)

**White:**

o Scottish

o Irish

o Other British

o Polish

o Gypsy/Traveller

o Any other white background

**Asian, Asian British or Asian Scottish:**

O Indian

o Pakistani

o Bangladeshi

o Chinese

o Any other Asian background

**Mixed / Multiple ethnic groups**

o Arab

o Any other Mixed/ Multiple ethnic background.

**Black/ Black Scottish / Black British/Caribbean**

o Black

o Black Scottish

o Black British

o Caribbean

o Any other Black/ Caribbean background

**African/ African Scottish/ African British**

o African

o African Scottish

o African British

o Other African

**Other ethnic group** (Please specify)

_____________________________________

**How long have you lived in the UK**

___________(Years) ___________(months)

Are you registered with a GP medical practice in North East Scotland?

o Yes

o No

Which university college and school does your course belong to?

(drop down menu)

**In which year of study are you?**

(drop down menu Undergraduate year 1, Undergraduate year 2 etc and postgraduate)

Have you ever had sexual intercourse?

- 1. Yes
  2. No

*Sexual Intercourse* includes all of the below:

**Vaginal sex** (penis-in-vagina intercourse); **Oral sex** (mouth-to-genital contact); **Anal sex** (penis-in-anus intercourse); **Fingering or hand jobs** (hand-to-genital contact)

Have you ever been tested for an STI?

- 1. Yes in past 6 months
  2. Yes but not in past 6 months
  3. No

What is an *STI?*

Sexually transmitted infections (also known as **STI**s, or STDs for ‘sexually transmitted diseases,’ or VD for ‘venereal diseases’) are infections that are commonly/have a high probability of being spread from person to person through **unprotected sexual intercourse**. Examples are Chlamydia, Gonorrhea, Syphilis, Herpes or HIV.

Have you had sexual intercourse in the past 6 months?

- 1. No
  2. Yes

How many sexual partners have you had in the last 6 months?

___________________

*Sexual Partners*

These include regular or casual partners that you have had sexual intercourse with.

*If answer 3b then:*

Please give a response to every question below

Have you...

| Had VAGINAL sex with a REGULAR partner without a condom? | Yes | No |
| --- | --- | --- |
| Had VAGINAL sex with a CASUAL partner without a condom? | Yes | No |
| Had ANAL sex with a REGULAR partner without a condom? | Yes | No |
| Had ANAL sex with a CASUAL partner without a condom? | Yes | No |

What is a *regular partner?*

Someone you have a regular relationship with and have sexual intercourse with e.g. boyfriend/girlfriend, husband/wife

What is a *casual partner?*

Someone you have sex with (once or several times), but with whom you have no regular

relationship.

**PART 2: PSYCHOLOGICAL CONTRUCTS**

Please indicate on the scale below your response to the following questions

| SUSCEPTIBILITY | Very Low |  |  |  | Very high |
| --- | --- | --- | --- | --- | --- |
|  | chance |  |  |  | chance |
| Considering your answers above and | 1 | 2 | 3 | 4 | 5 |
| what you know about STIs what do |  |  |  |  |  |
| you think your chances are of |  |  |  |  |  |
| contracting an STI? |  |  |  |  |  |
|  |  |  |  |  |  |
| Considering your answers above and | 1 | 2 | 3 | 4 | 5 |
| what you know about STIs what do |  |  |  |  |  |
| you think your chances are of |  |  |  |  |  |
| contracting Chlamydia? |  |  |  |  |  |
|  |  |  |  |  |  |
| Considering your answers above and | 1 | 2 | 3 | 4 | 5 |
| what you know about STIs what do |  |  |  |  |  |
| you think your chances are of |  |  |  |  |  |
| contracting Gonorrhoea? |  |  |  |  |  |
|  |  |  |  |  |  |
| Considering your answers above and | 1 | 2 | 3 | 4 | 5 |
| what you know about STIs what do |  |  |  |  |  |
| you think your chances are of |  |  |  |  |  |
| contracting Herpes? |  |  |  |  |  |

KNOWLEDGE

Please indicate whether you think each statement below is true, false or I don’t know. If you don’t know, please don’t guess, choose don’t know.

| 7) |  | True | False | Don’t |
| --- | --- | --- | --- | --- |
|  |  |  |  | Know |
| 1 | You can always tell if you have an STI because you would have |  |  |  |
|  | symptoms (e.g. changes in your body) |  |  |  |
| 2 | Symptoms of all STIs are painful |  |  |  |
| 3 | If you only have unsafe sex once, you will not get an STI |  |  |  |
| 4 | People who always use condoms are safe from all STIs |  |  |  |
| 5 | Some STIs can cause infertility in women (men?) |  |  |  |
| 6 | Not all STIs can be cured |  |  |  |
| 7 | Most STIs will go away on their own |  |  |  |
| 8 | Some STI’s can be treated with anitibiotics |  |  |  |
| 9 | You have to pay to get tested |  |  |  |
| 10 | If you don’t have symptoms (but want a test) the best place you |  |  |  |
|  | to get tested is at your local GP |  |  |  |

Which of the following can be tested for by taking a sample of your pee?

Please tick yes, no or I don’t know. If you don’t know, please don’t guess, choose don’t know.

|  |  | Yes | No | Don’t Know |  |
| --- | --- | --- | --- | --- | --- |
|  | Chlamydia |  |  |  |  |


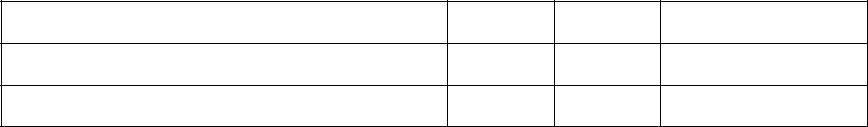


Gonorrhoea

HIV

Syphilis

SOCIAL NORMS

Please use the scale below to respond to the following statements

|  | Strongly |  |  |  | Strongly |
| --- | --- | --- | --- | --- | --- |
|  | disagree |  |  |  | agree |
| My sexual partners would want me to | 1 | 2 | 3 | 4 | 5 |
| be tested for STIs |  |  |  |  |  |
| My friends would want me to be | 1 | 2 | 3 | 4 | 5 |
| tested for STIs |  |  |  |  |  |
| My family would want me to be | 1 | 2 | 3 | 4 | 5 |
| tested for STIs |  |  |  |  |  |
| Doctors and health professionals | 1 | 2 | 3 | 4 | 5 |
| would want me to be tested for STIs |  |  |  |  |  |

*Sexual Partners*

These include regular or casual partners that you have had sexual intercourse with.

Please use the scale below to respond to the following statements

|  | Definitely |  |  |  | Definitely |
| --- | --- | --- | --- | --- | --- |
|  | not be |  |  |  | be tested |
|  | tested |  |  |  |  |
| If I knew my sexual partners wanted | 1 | 2 | 3 | 4 | 5 |
| me to get tested for STIs I would... |  |  |  |  |  |
| If I knew my friends wanted me to get | 1 | 2 | 3 | 4 | 5 |
| tested for STIs I would... |  |  |  |  |  |
| I I knew my family wanted me to get | 1 | 2 | 3 | 4 | 5 |
| tested for STIs I would... |  |  |  |  |  |
| If I knew my doctors and health | 1 | 2 | 3 | 4 | 5 |
| professionals wanted me to get |  |  |  |  |  |
| tested for STIs I would... |  |  |  |  |  |

Please use the scale below to respond to the following statements (if unsure, give your best guess)

|  | 0% |  | 25% | 50% | 75% | 100% |
| --- | --- | --- | --- | --- | --- | --- |
| What proportion of your friends have |  |  |  |  |  |  |
| ever been tested for STIs |  |  |  |  |  |  |
| What proportion of people your age |  |  |  |  |  |  |
| have ever been tested for STIs |  |  |  |  |  |  |
| What proportion of the general |  |  |  |  |  |  |
| population have ever been tested for |  |  |  |  |  |  |
| STIs |  |  |  |  |  |  |

ATTITUDES

*Direct*

*Please use the scales below to respond to the statement*

**Going to STI testing is...**

| Waste of time |  |  |  | Valuable |
| --- | --- | --- | --- | --- |
| 1 | 2 | 3 | 4 | 5 |
|  |  |  |  |  |
| Bad |  |  |  | Good |
| 1 | 2 | 3 | 4 | 5 |
|  |  |  |  |  |
| Painful |  |  |  | Painless |
| 1 | 2 | 3 | 4 | 5 |
|  |  |  |  |  |
| Interesting |  |  |  | Boring |
| 1 | 2 | 3 | 4 | 5 |
|  |  |  |  |  |
| Unwise |  |  |  | Wise |
| 1 | 2 | 3 | 4 | 5 |
|  |  |  |  |  |
| Harmful |  |  |  | Beneficial |
| 1 | 2 | 3 | 4 | 5 |
|  |  |  |  |  |
| Unappealing |  |  |  | Appealing |
| 1 | 2 | 3 | 4 | 5 |
|  |  |  |  |  |
| Unimportant |  |  |  | Important |
| 1 | 2 | 3 | 4 | 5 |
|  |  |  |  |  |
| Irresponsible |  |  |  | Responsible |
| 1 | 2 | 3 | 4 | 5 |
|  |  |  |  |  |

*Behavioural beliefs*

Please use the scales below to respond to the statement

**If I go to STI testing:**

| 13 | Unlikely/ |  |  | |  | |  | | | Likely/Agr |  |
| --- | --- | --- | --- | --- | --- | --- | --- | --- | --- | --- | --- |
|  | Disagree |  |  | |  | |  | | | ee |  |
| I will stay healthy | 1 |  | 2 | | 3 | | 4 | | | 5 |  |
| I will feel reassured | 1 |  | 2 | | 3 | | 4 | | | 5 |  |
| It will be embarrassing | 1 |  | 2 | | 3 | | 4 | | | 5 |  |
| I will get information and advice | 1 |  | 2 | | 3 | | 4 | | | 5 |  |
| It will be stressful | 1 |  | 2 | | 3 | | 4 | | | 5 |  |
| I will feel comfortable | 1 |  | 2 | | 3 | | 4 | | | 5 |  |
| It will negatively affect my/any future | 1 |  | | 2 | 3 | | 4 | |  | 5 | |
| relationships |  |  | |  |  |  |  |  |  |  | |
| I will feel responsible | 1 |  | | 2 | 3 | | 4 | |  | 5 | |
| It will be time-consuming | 1 |  | | 2 | 3 | | 4 | |  | 5 | |
| I’ll get the treatment I need before I | 1 |  | | 2 | 3 | | 4 | |  | 5 | |
| have complications |  |  | |  |  |  |  |  |  |  | |
| It will negatively affect my future and | 1 |  | | 2 | 3 | | 4 | |  | 5 | |
| career prospects |  |  | |  |  |  |  |  |  |  | |
| *Outcome evaluations* |  |  | |  |  |  |  |  |  |  | |
| Please use the scales to respond to each statement | | | |  |  |  |  |  |  |  | |
|  |  | | |  |  |  |  |  |  |  | |
|  | Not | | |  |  |  |  |  |  | Important | |
|  | important to | | |  |  |  |  |  |  | to me | |
|  | me | | |  |  |  |  |  |  |  | |
| To stay healthy is | 1 |  | | 2 |  | 3 |  | 4 |  | 5 | |
| To get reassurance is | 1 |  | | 2 |  | 3 |  | 4 |  | 5 | |
| To not be embarrassed is | 1 |  | | 2 |  | 3 |  | 4 |  | 5 | |
| To get information and advice is | 1 |  | | 2 |  | 3 |  | 4 |  | 5 | |
| To not feel stressed is | 1 |  | | 2 |  | 3 |  | 4 |  | 5 | |
| To feel comfortable is | 1 |  | | 2 |  | 3 |  | 4 |  | 5 | |
| To not negatively affect my/any | 1 |  | | 2 |  | 3 |  | 4 |  | 5 | |
| future relationships is |  |  | |  |  |  |  |  |  |  | |
| To be responsible is | 1 |  | | 2 |  | 3 |  | 4 |  | 5 | |
| To not be time-consuming is | 1 |  | | 2 |  | 3 |  | 4 |  | 5 | |
| To get the treatment you need before | 1 |  | | 2 |  | 3 |  | 4 |  | 5 | |
| you have complications is |  |  | |  |  |  |  |  |  |  | |
| To not negatively affect your future |  |  | |  |  |  |  |  |  |  | |
| and career prospects is |  |  | |  |  |  |  |  |  |  | |

SOCIAL FEAR

Please use the scales to respond to the statement

**If I had an STI...**

|  | Strongly |  |  |  |  | Strongly |  |
| --- | --- | --- | --- | --- | --- | --- | --- |
|  | disagree |  |  |  |  | agree |  |
| I would feel ashamed | 1 |  | 2 | 3 | 4 | 5 |  |
| I would feel embarrassed | 1 |  | 2 | 3 | 4 | 5 |  |
| People would avoid me | 1 |  | 2 | 3 | 4 | 5 |  |
| People would think badly of me | 1 |  | 2 | 3 | 4 | 5 |  |
| I would be worried about my parents | 1 |  | 2 | 3 | 4 | 5 |  |
| reaction |  |  |  |  |  |  |  |
| I would be worried about my sexual | 1 |  | 2 | 3 | 4 | 5 |  |
| partners reaction |  |  |  |  |  |  |  |
| I would be worried testing staff would | 1 |  | 2 | 3 | 4 | 5 |  |
| be gossiping about me |  |  |  |  |  |  |  |
| I would feel judged | 1 | 2 | | 3 | 4 | 5 | |
| SELF-EFFICACY |  |  | |  |  |  | |
| Please use the scales to respond to the statement | |  | |  |  |  | |
| **How confident are you to get an STI test if....** | | | |  |  |  | |
|  |  |  | |  |  |  | |
|  | Not at all | 25% | | 50% | 75% | Totally | |
|  | (0%) |  | |  |  | 100% | |
|  | confident |  | |  |  | confident | |
| The test site was far away | 0% | 25% | | 50% | 75% | 100% | |
| You had to go on own | 0% | 25% | | 50% | 75% | 100% | |
| You go with other people | 0% | 25% | | 50% | 75% | 100% | |
| If you had to wait for appt | 0% | 25% | | 50% | 75% | 100% | |
| If you knew someone there (taking | 0% | 25% | | 50% | 75% | 100% | |
| test/waiting room) |  |  | |  |  |  | |
| Even if you worry you might meet | 0% | 25% | | 50% | 75% | 100% | |
| someone you know |  |  | |  |  |  | |
| You think you are going to be older | 0% | 25% | | 50% | 75% | 100% | |
| than everyone else in the waiting |  |  | |  |  |  | |
| room |  |  | |  |  |  | |
| Not registered with a GP practice | 0% | 25% | | 50% | 75% | 100% | |
| I don’t have time | 0% | 25% | | 50% | 75% | 100% | |
| I’m not sure if a symptom is a | 0% | 25% | | 50% | 75% | 100% | |
| symptom of an STI |  |  | |  |  |  | |
| My friends have had bad experiences | 0% | 25% | | 50% | 75% | 100% | |
| I’ve had bad experiences in the past | 0% | 25% | | 50% | 75% | 100% | |
| If drop in sessions/appointments | 0% | 25% | | 50% | 75% | 100% | |
| were available at the weekend |  |  | |  |  |  | |
| If drop in sessions/appointments | 0% | 25% | | 50% | 75% | 100% | |
| were available in the evenings |  |  | |  |  |  | |
| If you could have the test posted to | 0% | 25% | | 50% | 75% | 100% | |
| you |  |  | |  |  |  | |
| If you could complete the test at | 0% | 25% | | 50% | 75% | 100% | |
| home |  |  | |  |  |  | |

INTENTION

Please answer the following question :

1. I intend to get a test for STIs in the next month?
   1. Yes
   2. No

Please indicate on the scale below your response to the following: I am planning to get an STI test in the next month?

| Totally disagree |  |  |  | Totally agree |
| --- | --- | --- | --- | --- |
|  |  |  |  |  |
| 1 | 2 | 3 | 4 | 5 |
|  |  |  |  |  |
